# Interfacial Interaction of Clay and Saturates in Petroleum-Contaminated Soil: Effect of Clay Surface Heterogeneity

**DOI:** 10.3390/molecules27227950

**Published:** 2022-11-17

**Authors:** Yang Yang, Xing Liang, Xiaobing Li

**Affiliations:** 1 National Center for Coal Preparation and Purification Engineering Research, China University of Mining and Technology, Xuzhou 221116, China; 2 School of Chemical Engineering and Technology, China University of Mining and Technology, Xuzhou 221116, China

**Keywords:** oil–clay interfacial interaction, adhesive force, rheology, adsorption heat, molecular simulation

## Abstract

Petroleum-contaminated soil (PCS) exhibits a variety of oil–soil interfacial properties. Surface heterogeneity of soil particles is one of the most critical influencing aspects. The interaction energies of the heterogeneous surfaces of montmorillonite (Mnt) and kaolinite (Kln) for saturates adsorption were determined by molecular simulation to be −1698.88 ± 0.67 (001 surface of Mnt), −73.81 ± 0.51 (010 edge of Mnt), −3086.33 ± 0.46 (001 surface of Kln), and −850.17 ± 0.74 (010 edge of Kln) kJ/mol, respectively. The adsorption of both clays with saturates relied on van der Waals forces, and the edges of Mnt were hardly adsorbed with saturates. According to adhesive force measurements, the oil–clay interaction forces of Mnt and Kln were 111.18 ± 0.01 and 122.65 ± 0.03 μN, respectively. In agreement with the simulations, Kln adsorbed saturates more strongly. Dynamic interfacial rheology and liquid viscoelasticity also revealed differences in adsorption behaviors between Mnt–saturates and Kln–saturates. It demonstrated that in the case of relatively low clay concentrations, the impact of particle surface heterogeneity on the adsorption process was stronger than that of structure even though Mnt had multilayer structures. Moreover, in thermodynamic adsorption experiments, it was evident that Kln adsorbed more oil than Mnt at the adsorption equilibrium states even though both were multilayer adsorptions and the adsorption amounts declined with increasing temperature. Simultaneously, the characteristics of the thermal adsorption of clay and saturates with different proportions were consistent with clay dispersion in saturates, and Kln released more heat being combined with oil. Overall, the heterogeneity of clay particles strongly affects the oil–clay interfacial chemical behaviors, causing more difficulty in treating PCS containing Kln than those containing Mnt. These results provide a theoretical basis for PCS treatment technology.

## 1. Introduction

Oil enters soil during the exploitation, refining, storage, and transportation processes, which results in petroleum-contaminated soil (PCS) [1]. When the pollution exceeds the self-purification of the soil, the regular function of the soil ecosystem is disrupted, and soil quality declines [2]. Toxic and hazardous aliphatic, alicyclic and aromatic hydrocarbons, halogenated hydrocarbons, sulfur compounds, and nitrogen compounds are the primary pollutants released by crude oil into the soil, and they reduce the variety of plants and microorganisms in the soil and deplete soil fertility [3]. Worse yet, after being absorbed by plants and animals, such pollutants accumulate and travel down the food chain, putting human health at risk by causing dermatitis, gastrointestinal illnesses, fetal abnormalities, malignant tumors, and other diseases that are increasing rapidly [4]. As a result, restoring soil function through non-hazardous soil remediation techniques is essential for conserving the entire ecosystem [5].

Numerous methods have been developed for the treatment and remediation of PCS. The following technologies are frequently applied: extraction [6], pyrolysis [7], biological treatment [8], and water-washing treatment [9]. However, the effects of the same treatment approach applied to different polluted soils show significant variation. For instance, when oil sands were cleaned using water washing, the asphaltene was easily removed from the silica surface by the action of chemicals and fluid disruption. In contrast, asphaltenes on clay surfaces were difficult to remove [10]. The difference between properties of oil–silica and oil–clay interfaces is the fundamental reason for this variation [11]. Therefore, remediation of PCS begins by recognizing the complicated interaction of oil–soil [12]. However, the understanding of oil–soil interfacial properties is still very limited, which makes it hard to guide the improvement of the remediation techniques for PCS.

The composition of PCS is quite complicated and contains minerals with different surface properties that significantly affect the oil–solid interaction [13]. For example, based on the high adsorption capacity of the large specific surface area, it is possible to infer that illite (specific surface area of 65–100 m^2^/g) adsorbs more oil than kaolinite (specific surface area of 5–30 m^2^/g) [14,15]. However, the opposite oil absorption results were confirmed for the two minerals, proving that the differences in adsorption capacity were actually caused by varying degrees of mineral wetting [16]. Furthermore, it has been proposed that mineral surface heterogeneity complicates surface wetting behavior [17]. Mineral surface heterogeneity occurs because the same mineral inevitably exposes surfaces of different chemical compositions due to varying fracture angles [18]. As a result, it is essential to consider the effect of mineral surface heterogeneity on oil–soil interactions rather than predicting the results of oil adsorption based on mineral structure alone. For example, Mnt and Kln belong to minerals with high abundance in clays. They both have two basal surfaces: the tetrahedral siloxane surface exposed by Si-O-Si and the octahedral aluminum surface exposed by Al-OH [19]. The structure is disrupted, and fractures occur at the edges of the layers, which are then compensated with -OH groups [20]. Although the two clays have dramatically differing adsorption of crude oil, the direct visualization of the spatial distribution of crude oil on mineral surfaces is lacking, and the effect of heterogeneous mineral surfaces on oil adsorption is unclear.

In addition to the mineral surface properties, the type of oil is also an essential factor influencing the oil–soil interface interaction [21]. To facilitate research and technical applications, crude oil components are usually divided into saturates, aromatics, resins, and asphaltenes depending on their polarity and solubility in different organic solvents [22]. Among them, asphaltene has attracted a lot of investigation due to its high polarity since it is considered the main factor affecting the surface properties of soil minerals [23]. However, saturates constitute the highest proportion (64.34% of the weight) of crude oil, and they have great migratory ability, which allows them to easily spread to deep soil levels and widens the scope of oil pollution [24,25]. For example, the analysis of PCS around well 6# in the Daqing oilfield revealed that at a depth of above 20 cm, hydrocarbons accounted for more than 90% of the organic matter, with saturates accounting for over 60% [26]. Therefore, the study of the relationship between saturates and soil minerals should not be neglected. 

This study aimed to investigate the effect of clay surface heterogeneity on saturates−clay interfacial properties. Oil−clay interactions were quantified by simulating the corresponding motion processes, calculating the interaction energies, testing the adhesive forces, investigating the rheological properties of the clay−oil suspensions, and measuring the adsorption heat and the adsorption amount.

## 2. Results and Discussions

### 2.1. Rheological Characterization of Saturates–Clay Suspensions

#### 2.1.1. Dynamic Oscillations of Saturates with Clay

**Figure 1:**
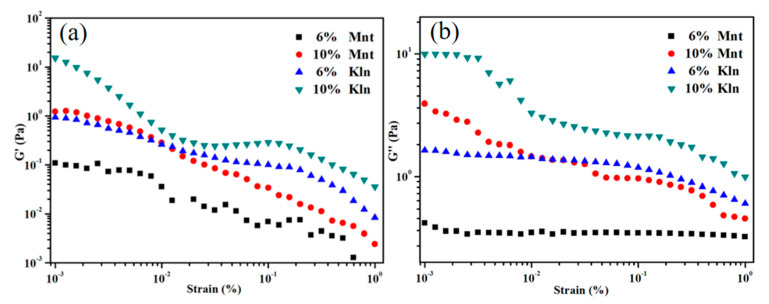
Strain sweep tests on storage (**a**) and loss modulus (**b**) of clay−oil dispersions containing 6% and 10% *w*/*w* clay in saturates at 6.28 rad/s frequencies.

In order to illustrate the stability of the two oil–clay mixtures, rheological tests were carried out. From the suspensions with clays shown in Figure 1, strain thinning behavior is evident, as the strain shows an increasing trend regardless of particle concentration. Compared with Mnt suspensions, the suspension containing Kln displayed a plateau in the storage modulus (G′), indicating that the clays formed an interface throughout the mixture, which prevented phase separation in a certain morphology [27]. Sequentially, increasing shear stress destroyed the created structure, which led to instability. On the other hand, the suspension including Kln had a more complicated flow pattern, and the nonlinear variation of G′ and G′′ indicated that more dynamic structural changes occurred as a result of the deformation. It was possible that the Kln surface interacted more strongly with the oil than the Mnt surface, resulting in a more stable oil–clay interface and higher compatibility between oil and Kln [28]. In addition, G′′ in Figure 1b for the Mnt suspensions are higher than G′ in Figure 1a, indicating that liquid-like characteristics are superior to solid-like characteristics. This was the opposite for Kln, which exhibited better solid-like characteristics.

**Figure 2:**
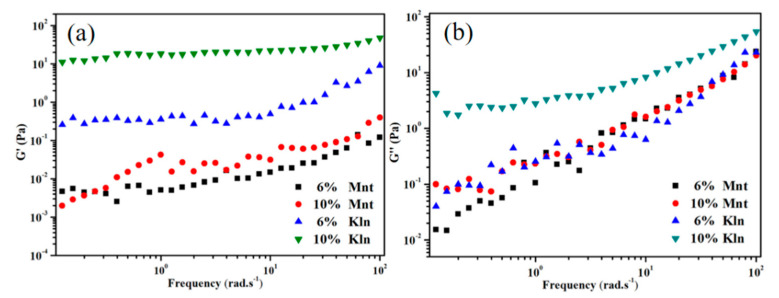
Frequency sweep tests on storage (**a**) and loss moduli (**b**) of four clay–oil dispersions containing 6% and 10% *w*/*w* clay in saturates at a 0.03% strain.

An additional investigation into the influence of clay on suspension stability via oscillatory frequency sweeps is presented in Figure 2, where the elastic and viscous parts of the system are denoted by G′ and G′′, respectively. It should be noted that the upward trend of G′′ in Figure 2b was more obvious in the high-frequency range, indicating that the frequency dependence of G′′ was more sensitive than those of G′. The 10% Kln suspensions exhibited G′ values that remained essentially unchanged in the high-frequency region compared with the low-frequency region, suggesting stronger solid-like properties that were consistent with the previous sedimentation results. This is because Kln disperses well in oil due to the chemical affinity between the heterogeneous surfaces and the oil. Interestingly, the G′ values for Mnt were lower than its G′′ values, while Kln displayed the opposite results. This indicates potentially stronger fluidity of Mnt suspensions [29]. Moreover, as the amount of Mnt increased, the viscous modulus of Mnt remained greater than its elastic modulus. This difference between Kln and Mnt was likely due to the low compatibility of some Mnt heterogeneous surfaces with oil and the weak van der Waals force in general, resulting in uneven dispersion of mixed phases. Combining all the experimental data, the conclusions are that the high viscoelasticity and stability of the suspensions are determined by the strength of the force between the surface of the heterogeneous surface and the oil. The suspension is more stable with a large van der Waals force of oil–clay interactions.

#### 2.1.2. Interfacial Rheology of Saturates and Clay

**Figure 3:**
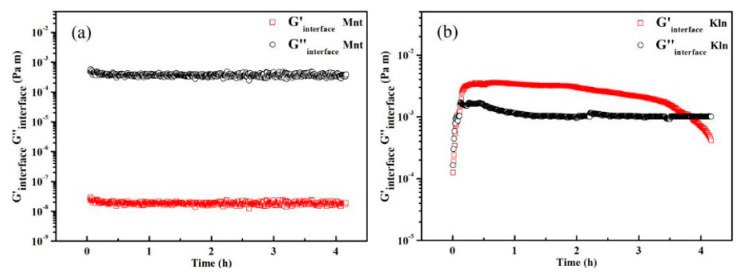
Oscillatory time sweep tests of clay–oil interface with 3000 ppm (**a**) Mnt and (**b**) Kln dispersed in saturates at 298 K.

The abovementioned device used to analyze the interfacial shear rheology of the samples is displayed in Figure S1 (Supplementary Information). The results for Kln showed that the interface exhibited an increased interfacial storage modulus due to particles adsorbing at the saturates interface and forming an interfacial layer (G′_interface_). Figure 3b clearly shows that Kln produced larger interfacial moduli (G′_interface_ and G′′_interface_) than Mnt, implying that particles accumulated more at the interface, resulting in a denser and stronger interfacial layer. Apart from a small G′′_interface_ value, the Mnt interface did not respond to the interfacial rheology test, thus demonstrating that the Mnt did not undergo adsorption in the oil interface (Figure 3a). Despite its multilayered structure, Mnt did not initially form an interface with oil due to the small van der Waals force of the Mnt–oil interface. The shear force directly destroyed the clay–oil layer at the interface. Therefore, it could be concluded that the features of the particle surface had a higher impact on the adsorption process than the structure with a relatively small amount of clay. In contrast, Kln took a long time to form a weak interfacial layer, with the G′_interface_ exceeding the G′′_interface_ at approximately 12 min, thus indicating viscoelastic behavior after loading. The G′_interface_ and G′′_interface_ values were significantly higher for Kln than Mnt, indicating that particles underwent strong interaction with oil and overcame gravity, forming a cohesive interfacial layer [30]. With the continuous wetting of Kln particles, the G′_interface_ value gradually decreased, indicating a reduction in the aggregation of particles at the interface as well as a decrease in stability and uniformity. The adhesive force between the clay surface and oil remained unchanged when the clay was thoroughly wetted. However, the particle–oil layer outside would adsorb more oil droplets. The oil molecules were not uniformly distributed in the clay area of a particular region, resulting in the aggregation of oil droplets containing clay [31]. Moreover, their gravity increased as particles were covered in multilayer oil, reflecting the fact that the particles were impacted by gravity and exited the interface to enter the oil phase. This conclusion suggests that the existing surface heterogeneity of clay leads to differences in clay–oil interfacial stability and provides an intuitive basis for investigating surface adsorption processes.

### 2.2. Thermodynamics Adsorption Analysis of Saturates on Clay

#### 2.2.1. Adsorption Isotherms

**Figure 4:**
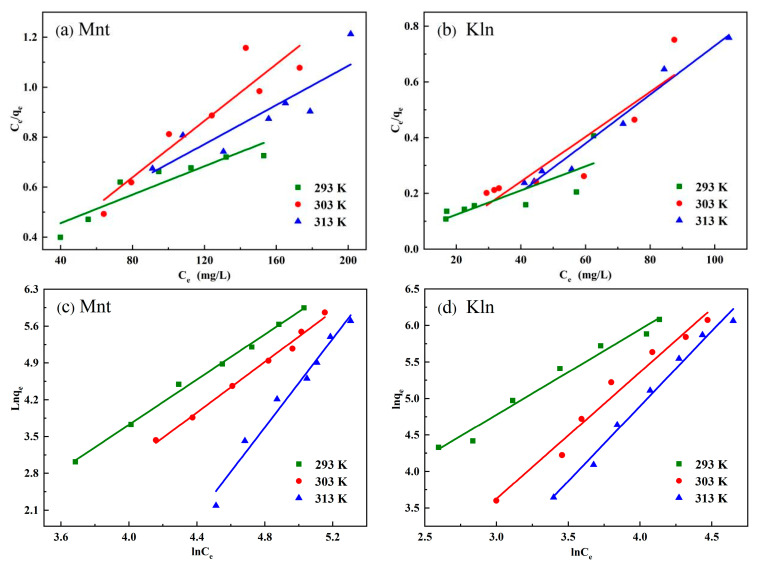
The Langmuir model fitting curves of (**a**) Mnt and (**b**) Kln, and the Freundlich model fitting curves of (**c**) Mnt and (**d**) Kln with clay dosage of 0.05 g·L^−1^ at 293, 303, and 313 K.

In order to compare the surface adsorption processes of Mnt and Kln under natural environmental conditions, the isothermal adsorption of clays on saturates was determined. As shown in Figure S2a and Figure 2b, the equilibrium adsorption *q_e_* increases with the increase in initial oil concentration *C*_0_ until it reaches the maximum. Two common adsorption isotherm models, the Langmuir isotherm and the Freundlich isotherm, were employed to analyze the adsorption isotherm data as follows:

(1) $$\frac{C_{e}}{q_{e}}=\frac{1}{K_{l}q_{m}}+\frac{C_{e}}{q_{m}}$$

(2) $$\ln q_{e}=\frac{1}{n}\ln C_{e}+\ln K_{f}$$

where *q_e_* (mg/g) is the amount of oil absorbed per unit mass of clay and *C_e_* (mg/L) is the oil concentration at equilibrium. *q_m_* (mg/g) represents the maximum adsorption capacity. *K_l_* and *K_f_* denote Langmuir and Freundlich isotherm constants, respectively, and 1/*n* is the heterogeneity factor of the adsorbent. Figure 4 depicts the two model fitting curves for Mnt–saturates and Kln–saturates, and Table 1 lists the specific parameters. 

Figure S2a and Figure 2b indicate that Kln adsorbs more oil than Mnt, which is consistent with rheological studies in this paper showing stronger adhesion between Kln and oil. More clearly, as the initial oil concentration increased, both Mnt and Kln had no maximum adsorption capacity at the same temperature, indicating that the saturates exhibited multilayer adsorption on both clays and that the quantity of adsorption decreased with increasing temperature. Table 1 shows that oil absorption onto clay is an exothermic process. Hence, raising the temperature was not conducive to the adsorption of oil on both clays. Subsequently, at 293, 303, and 313 K, the correlation coefficient *R*^2^ values and constants obtained by the two isotherm models of the two clay-absorbed saturates are shown in Table 1. The *R*^2^ values of the Freundlich model were found to exceed 0.97 at three temperatures, indicating the Freundlich model could describe the adsorption of saturated fractions in both clays well. This also demonstrated the multilayer adsorption of saturates on both clays. The measuring standard for adsorption strength or surface heterogeneity was the slope 1/*n* between 0 and 1. A value near 0 indicated that the particle surfaces became more heterogeneous after oil adsorption. Under the complete adsorption condition, oil appeared to cover a larger area of Kln than Mnt, which might be partially uncovered by oil, demonstrating that the heterogeneity of the clay surface was important for saturates adsorption [32].

#### 2.2.2. Adsorption Heat Analysis

The level of heat produced at adsorption can also reflect the intensity of oil adsorbing on the clay surfaces. These values are presented in Table 2, where the minus sign indicates an exothermic process with spontaneous adsorption. The adsorption heat values for Mnt at different concentrations (−11.957 J/g for 0.6% *w*/*w* and −14.029 J/g for 1.0% *w*/*w*) were relatively small when the clay particles were in sufficient contact with the oil, and the equilibrium time increased with the increase of heat (112 min and 145 min, respectively). In comparison, the time required to achieve adsorption heat equilibrium with various concentrations of Kln (73 min and 90 min for 0.6% *w*/*w* and 1.0% *w*/*w*, respectively) was lower, as was the relatively large amount of heat produced during that period (−15.333 J/g and −24.985 J/g for 0.6% *w*/*w* and 1.0% *w*/*w*, respectively). These high heat values can be explained by the existence of a heterogeneous surface in Kln, indicating that each surface exposed to particles can adsorb oil molecules within a short time of being completely wetted. In comparison, Mnt contains areas that cannot adsorb oil molecules and might require a long time to interact with the oil to improve compatibility [33]. These results were also consistent with the interfacial moduli results. As a result, the adsorption heat and stabilization efficiencies were shown to be closely related, as higher adsorption heats result in oil–clay interactions with more stable adsorption and more unfavorable conditions for separation. Considering all the experimental data, the clay particle surface heterogeneity had a significant impact on the amount of saturates adsorbed on the clay. However, further experiments are needed to understand the microscopic mechanisms at the interface of the oil–clay interaction.

### 2.3. MD Simulation Analysis

#### 2.3.1. Conformational Analysis of Clay Adsorption Saturates Process

**Figure 5:**
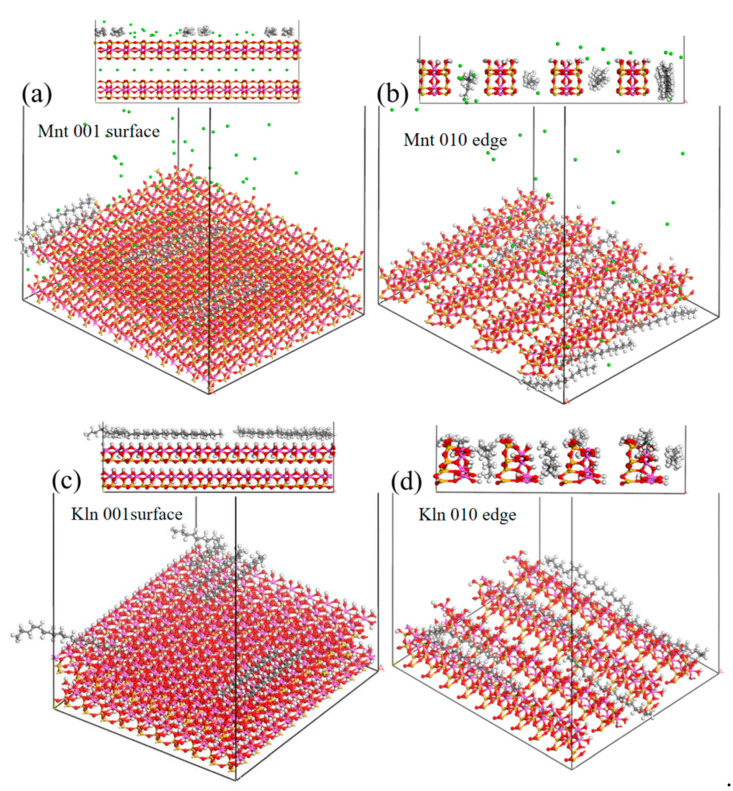
Equilibrium configuration structures of saturates adsorbed on (**a**) 001 surface and (**b**) 010 edge of Mnt and (**c**) 001 surface and (**d**) 010 edge of Kln at 298 K.

To investigate the action trajectory of saturates molecules on clay surfaces, the equilibrium adsorption conformations from molecular dynamics simulations are presented in Figure 5. These simulations visualized the site of action of the saturates adsorbing tightly on both clays. However, both Mnt models had flat and uniform adsorption only on the 001 surface. There were virtually no oil molecules at the 010 edge position, and the oil moved into the gaps, where it was tightly and uniformly adsorbed on the 001 surface shown in Figure 5b. Although most of the oil moved into the gap at the 010 edge of Kln, some were also adsorbed on the 010 edge, as seen in Figure 5d. Further, the interaction energies between the saturates and the edge and surface of the two clays were calculated. Since the saturates were typically nonpolar molecules with no electrostatic force, the total interaction energies were provided by van der Waals forces with values of −1698.88 ± 0.67, −73.81 ± 0.51, −3086.33 ± 0.46, and −850.17 ± 0.74 kJ/mol, corresponding to the 001 surface of Mnt, 010 edge of Mnt, 001 surface of Kln, and 010 edge of Kln, respectively. It was clear that the van der Waals forces of the saturates on the 001 surface of the clay were much greater than those on the 010 edge regardless of the clay type. Noting that the van der Waals force on the 010 edge of Mnt was far less than those on the 001 surface, it could be speculated as to the reason that the Mnt with heterogeneous surfaces had little contact with oil on its edges. Moreover, not only was the van der Waals force between Kln and oil on the 001 surface greater than that of Mnt, but there was also a strong van der Waals force at the 010 edge of Kln, indicating a strong interaction between saturates and Kln. In fact, the probability of exposing the 001 surface of a naturally broken clay is greater than that of exposing the 010 edge because breaking interlayer van der Waals forces and hydrogen bonds is considerably easier than breaking chemical bonds [34]. Thus, for Mnt and Kln particles with heterogeneous surface properties, there is better affinity of saturates for Kln than Mnt.

#### 2.3.2. Analysis of Saturates Distributions on Surfaces and Edges of Clay

**Figure 6:**
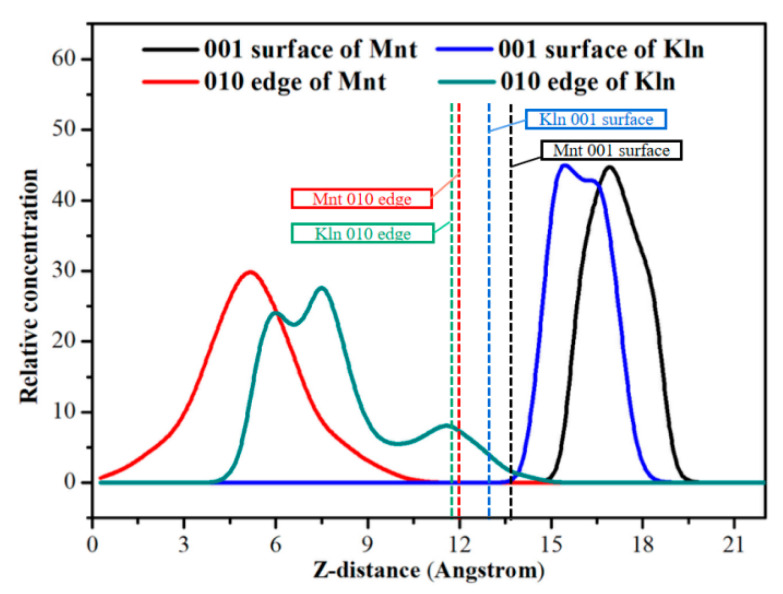
Relative concentration profiles of saturates molecules in surfaces and edges of Mnt and Kln along the *Z*-axis at 298 K.

Figure 6 depicts the relative concentration distribution of saturates adsorption on the clay surface along the *Z*-axis to observe the spatial location of saturates in two clay systems after a 1000 ps simulation. Between 0 and 14 Å existed in the location of four models. The adsorption range of oil on the Mnt 001 surface was 15.4–19.4 Å, while the adsorption range on the corresponding Kln surface was 13.6–18.7 Å. This difference might be due to the higher van der Waals force between Kln and saturates, resulting in the oil being closer to the Kln 001 surface and adsorbed more tightly (the oil distance from the Kln 001 surface was 0.8 Å and from the Mnt 001 surface was 1.4 Å). For the adsorption on the 010 edge of Mnt, it was obvious that oil molecules were distributed inside the model with a range of 0.2–10.8 Å. However, the oil distribution span in Kln for the edges and surfaces was 4.4–14.7 Å. The relative concentration of oil on the edges was lower than that on the gaps, indicating that saturates were more inclined to adsorb on the 001 surface present in the gaps. Furthermore, for Mnt and Kln particles with heterogeneous surface properties, the 010 edges of both clays exhibit a scattered distribution range and lower relative concentrations, indicating that saturates have a tendency to adsorb to the 001 surface and confirming the previous observations reported in Figure 5b,d.

### 2.4. Interaction Forces between Clay and Saturates

**Figure 7:**
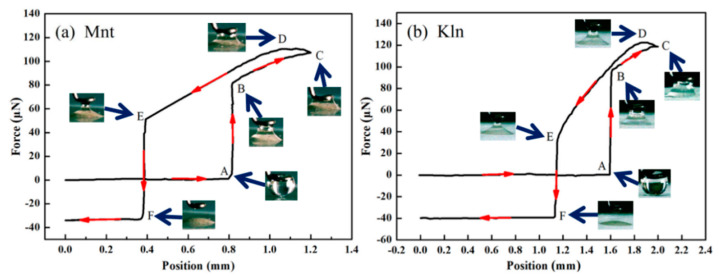
Force profiles and images of (**a**) Mnt and (**b**) Kln interacting with oil droplets at 298 K.

Following a sufficient understanding of the oil–clay interaction in the ideal state, the actual adhesive forces between oil and clays were tested. The force profiles for an oil droplet (saturates) interacting with the tablet surface of clay particles are depicted in Figure 7. Each test started with a zero net force and defined downward force as a positive value. The plate was lifted to the previous moment of contact with the oil droplet, which is marked as position A. When the oil droplet touched the clay plane, the force sharply and instantaneously increased to position B. The height of the tablet position is almost the same as position A. The force measured at position B is the snap-in force (F_in_) and is the maximum spreading force [35]. The dramatic increase in force was accompanied by the wetting area spreading of the droplet, reflecting high adsorption between saturates and clay. Compared with Mnt, there was a higher force between Kln and saturates. Between the distances labeled position B and C in Figure 7a,b, with the clay plane rising 0.4 mm, the oil droplet continued to diffuse and wet on the clay surface due to the increased adhesive force. Meanwhile, the oil droplet was squeezed between the stationary ring and the rising plane, generating a force to resist the compression of the oil droplet. As a result, the overall force increased at a slower pace. Compared with Kln, sluggish diffusion of saturates on the Mnt surface and high forces of oil droplet to resist compression resulted in Mnt having a lower resultant force than Kln. Point C was the highest position restricting plate elevation, after which the clay plane would return to its original position. Even though the oil droplet on the ring continued to adsorb on the clay surface, the resistance of oil droplet compression gradually decreased, leading to an increase in total force. When reaching position D, where it was at its maximum (F_max_), the force of the oil droplet resisting compression vanished and only the van der Waals force between oil and clay remained to provide the total force [36]. This maximum force was representative of the adhesion between the saturates and the clay surface. As the plate receded from the ring after position D, the oil droplet generated an increasing tensile resistance, causing the total force to eventually decrease. From position E, the adhesive force gradually decreased as the droplet began to detach from the ring, and position F was the point of complete disengagement where a negative force called the pull-off force (F_off_) occurred. The strong van der Waals force of adsorbed oil on Kln edges and surfaces was what distinguished it from the Mnt. This was consistent with simulation results, which showed that positions A through F for Kln had a greater distance than for Mnt (Figure 7a,b). The value of the negative force represents the mass of the oil droplets leaving the ring in Figure 7a (−33.95 ± 0.01 μN) and Figure 7b (−40.12 ± 0.02 μN), meaning that the mass of saturates remaining on the ring was enhanced by the increase in the oil–clay adhesive force. These observations indicate that the van der Waals forces generated by the clay edges and saturates have significant impact on oil adhesion behavior, especially for Kln and Mnt clays.

Table S1 summarizes the measured forces for the Kln and Mnt clay samples. It was clear that F_in_, F_max_, and F_off_ were all higher for Kln than for Mnt, indicating an intimate connection between these values and the surface heterogeneity of the clay (Kln: F_in_ = 96.69 ± 0.02, F_max_ = 122.65 ± 0.03, F_off_ = 32.68 ± 0.06 μN; Mnt: F_in_ = 80.87 ± 0.03, F_max_ = 111.18 ± 0.01, F_off_ = 22.72 ± 0.05 μN). These differences were expected since the heterogeneous surface of Kln produces oil–clay van der Waals forces when combined with oil, resulting in higher adhesion between oil and Kln. These trends suggest that compared with Mnt, the saturates mixed with Kln were more resistant to being removed. This conclusion is consistent with the rheology and adsorption test results that indicate that the existing surface heterogeneity of clay leads to differences in adhesive force at the oil–clay interface and provides an intuitive basis for investigating the strength of oil–clay interactions.

## 3. Materials and Methods

### 3.1. Materials and Preparation of Clay Dispersions

Natural Mnt and Kln were purchased from Sinopharm Chemical Reagent Co. Ltd. (Shanghai, China), with particle sizes ranging from 2.9 to 16.6 μm. The structures are shown in Figure S3. In this study, paraffin was used to represent the saturates in crude oil. The density was 0.84 g/cm^3^, and the viscosity was 76.51 Pa∙s at 20 °C. To prepare suitable suspensions, the clay powders with concentrations of 6% and 10% *w*/*w* in oil were selected, as shown in Figure S4. To ensure that the clay particles were completely wetted, they were mixed with the oil phase at room temperature and magnetically stirred for 4 h. The suspension stabilities in this study were based on sedimentation experiments conducted over 30 min after uniform dispersions were created, as shown in Figure S4d–f. The duration of these settlement tests was consistent with the time required for rheological measurements.

### 3.2. Adhesive Force Measurements

**Figure 8:**
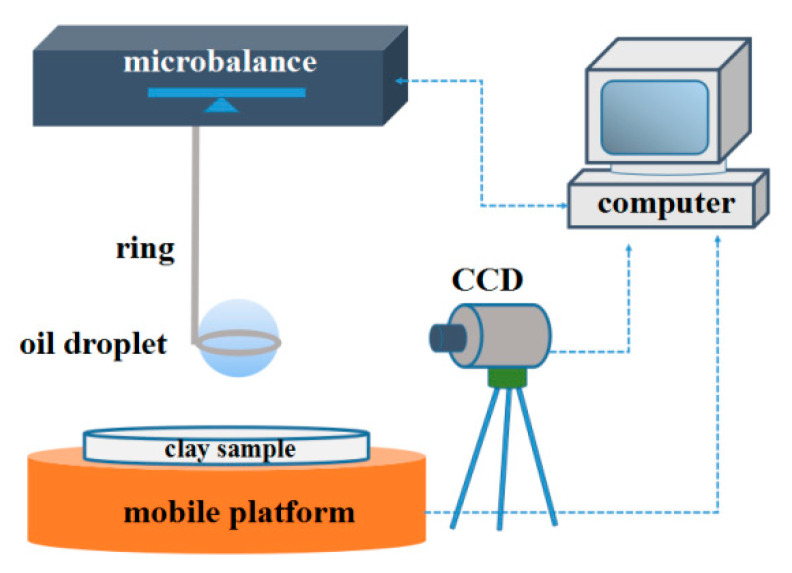
Microbalance experimental setup used during adhesion between oil droplets and clay.

Differences in the interactions between saturates and clay particles were measured by a microelectronic mechanical balance, which was modified and constructed on the basis of JK99M2 (Powereach, Shanghai, China). Figure 8 depicts the setup for Adhesive measurements. During testing, an approximately 2 μL oil droplet was suspended on a platinum ring linked to the microbalance. Separately, clay particles were pressed into a flat, rigid sheet with 0.5 cm thickness and then immobilized horizontally on a lifting shelf, which was controlled to move up or down toward or away from the oil droplet at a rate of 0.01 mm/s. The forces and masses of the ring with saturates were measured and then set to zero before every measurement.

### 3.3. Rheological Measurements

Rheological properties of suspensions were assessed using an MCR 302 rheometer by Anton Paar, Graz, Austria. It was equipped with a parallel plate fixture (50 mm diameter) for dynamic oscillatory sweep tests to measure viscoelastic parameters of strain with frequency sweeps involving storage modulus G′ (Pa) and loss modulus G′′ (Pa). Strain amplitude sweeps for all suspensions were conducted at a constant angular frequency (ω) of 6.28 rad/s in the varying strain (γ) region of 0.1% to 100%. Dynamic frequency sweeps were also accomplished using angular frequencies (ω) from 0.05 to 10 rad/s at a constant strain (γ) of 0.03%. To obtain repeatable results, each dispersion was mechanically stirred with a vortex mixer for 10 min before testing. The interfacial shear rheology of the interfacial adsorption layer was investigated by an oscillatory time sweep over 250 min (γ = 0.1%, ω = 1 s^−1^). It was equipped with biconical geometry, and all tests were conducted at 20 °C.

To examine the adsorption characteristics of the clay at the saturates interface, interfacial shear rheology experiments were performed on both Kln and Mnt clay samples. The device used for interfacial shear rheology displayed a bottom-liquid phase of pure paraffin reserved for tests and a rotor stopped at the oil–air interface. The top-liquid phase was injected with clay fully dissolved in ethanol, which was insoluble in paraffin. The interfacial structure of the clay–oil layers was fragile, as it was damaged by shear force and gravity and caused low interfacial moduli (storage modulus G′_interface_ and loss modulus G′′_interface_) [37]. For this reason, a concentration of 3000 ppm of clay was selected to disperse on the surface of the oil phase. The interactions between the air and clay after the ethanol evaporated were ignored for this experiment. Each of these tests was conducted over four hours with oscillatory time sweeps.

### 3.4. Adsorption Heat Measurements

Adsorption heat measurements between the clay samples and saturates were performed using microcalorimetry on a C80 calorimeter by Setaram, Lyon, France. Both samples were taken as 7 mg and 10 mg and 2 mL of the liquid phase placed at the bottom and upper parts of a reversal container, respectively. The two substances were then mixed by turning over the container. The heat flux curves were integrated to calculate adsorption heat.

### 3.5. Thermodynamic Adsorption Measurements

For the saturates adsorption test, 0.05 g of clay was added in a series of 50 mL bottles, and the initial concentration of saturates was 100~500 mg L^−1^. These bottles were placed in a bath and shaken to achieve adsorption equilibrium at selected temperatures (293, 303, and 313 K). The adsorption capacity (*q_t_*) of the clay was determined by the equation of *q_t_* = *V·*(*C*_0_ − *C_t_*)/*m*, where *q_t_* (mg·g^−1^) is the adsorption capacity, *V* (L) is the volume of saturates in water emulsion, *m* (mg) is the weight of clay, and *C_t_* (mg L^−1^) is residual oil content.

### 3.6. MD Simulation Details

**Figure 9:**
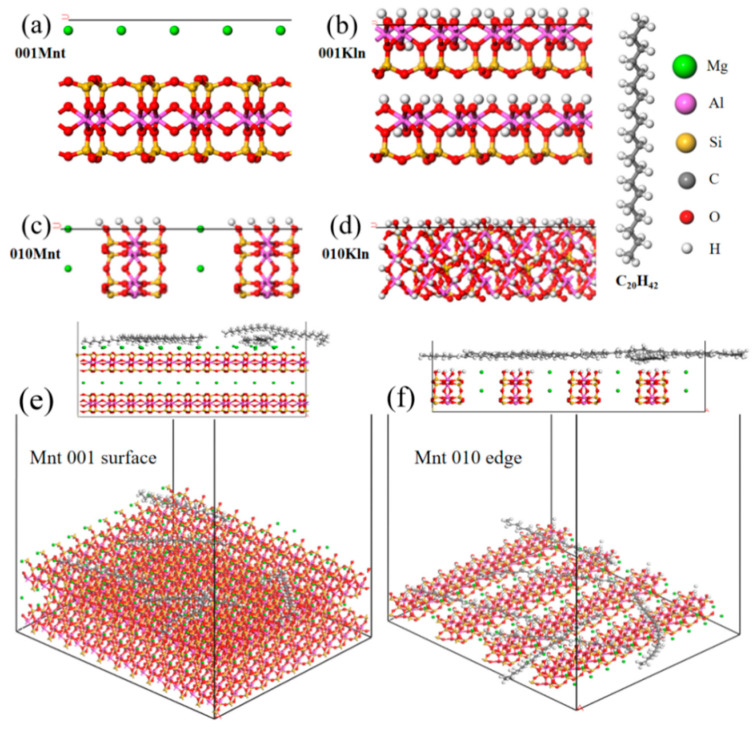

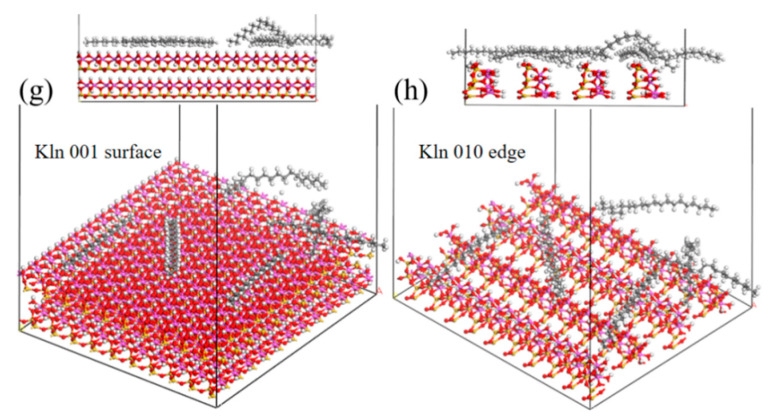
Molecular structures of (**a**) 001 surface and (**c**) 010 edge of Mnt, (**b**) 001 surface and (**d**) 010 edge of Kln and saturates (C_20_H_42_), and initial configuration structures of saturates adsorbed on (**e**) 001 surface and (**f**) 010 edge of Mnt and (**g**) 001 surface and (**h**) 010 edge of Kln in 298 K.

The molecular motion process was simulated by Materials Studio (MS) 8.0 software (Accelrys, Inc., San Diego, CA, USA). As displayed in Figure 9, models of Mnt, Kln, and saturates (selecting C_20_H_42_, which has been widely used in modelling as a representative compound of saturates) were created [21]. Cutting the 010 edge and 001 surface of the clay molecules cell yields unsaturated atoms such as Si-O or Al-O that are truncated and suspended on the 010 edge model. Still, there is no unsaturated atom of the truncated bond on the 001 surface. Referring to the recognized methods of neutralizing unsaturated atoms, a H atom (forming an -OH group) was added to the non-bridged O, and an -OH group was added to the three-coordinated Si [38,39]. Although it is known that the charge density on the clay 010 edge varies with pH, it allows the original, empirically obtained crystal structure to relax and adsorb organic matter rather than to account for this explicitly [40]. Because saturates are nonpolar chemicals, pH has no influence on them in the simulated force field [41]. The supercell was used to extend the model along the section once it had been created. It was worth mentioning that a larger surface was required to guarantee complete saturates adsorption and expansion to a section of around 60 × 50 Å. After placing six saturates molecules on the surface, a vacuum layer of 100 Å was installed on the models to remove the periodic effect [42]. Subsequently, the intelligent method was used for geometrical configuration optimization of the three-dimensional models to reduce the energy of the system with the parameters shown in Table S2. Based on established models, the clay interface force field (ClayFF) [43] and the canonical ensemble (NVT) [44] were utilized to calculate the foresight of the model. The total energies of long-range electrostatic interaction and bondless van der Waals interactions were calculated using atom-based methods with a 15.5 Å radius cutoff. The 1000 ps [45] dynamic calculation of the system was performed at 298 K and 0.5 fs time steps, while the energy and temperature convergence curves were monitored to guarantee system balance.

## 4. Conclusions

In this paper, we investigated the oil–clay interface interactions of PCS concerning clay surface heterogeneity. At the macroscopic level, Kln and Mnt mixtures containing saturates were investigated along with the adsorption behavior of the saturated fraction on their surfaces. A difference in heat release during oil adsorption by the two clays was also evident by the adsorption heat. These results were caused by the heterogeneous surfaces of Kln and Mnt. The relationship between interfacial forces and interaction energies between Kln and Mnt and saturates were studied from microscopic perspectives, which verified the conclusions obtained from macroscopic experiments. Because of the large van der Waals forces and interaction energies generated between the surface and edges of Kln and oil, the adhesive force between Kln and oil was greater than that of Mnt. Conversely, van der Waals forces and interaction energies between the edges of Mnt and oil were very fragile. Only its surface generated relatively strong forces and energies with the oil. Although Mnt had multilayer structures, the clay surface heterogeneity had a stronger effect on the interaction process at the oil–clay interface than the structure. Thus, the heterogeneous surfaces of Kln were better at adsorbing oil than those of Mnt. Overall, the interfacial behavior of oil–clay interfaces was determined by the interaction between oil and heterogeneous clay surfaces. 

Despite only investigating Mnt and Kln, which are representative of clay, and one of the highest concentration components of petroleum, the research mechanism outlined in this study can still serve as a basis for developing better practices in the decontamination of PCS.

## Figures and Tables

**Table 1:** Isothermal model of saturates adsorption on clay surfaces derived from Langmuir and Freundlich model.

Type	T (K)	Langmuir			Freundlich		
		*q_m_* (mg/g)	*K_l_* (L/mg)	*R_l_* ^2^	*K_f_*	1/*n*	*R_f_* ^2^
Mnt	293	230.95	0.116	0.6037	35.52	0.615	0.9921
	303	124.22	−0.101	0.7874	31.48	0.407	0.9710
	313	114.29	−0.060	0.9551	22.04	0.251	0.9828
Kln	293	350.88	0.008	0.8273	50.34	0.721	0.9948
	303	255.10	0.030	0.8494	42.17	0.559	0.9905
	313	176.68	0.013	0.7459	36.11	0.381	0.9895

**Table 2:** Heats of adsorption with different equilibration times for the different concentrations of clays with saturates at 298 K.

Clay (*w*/*w*)		Adsorption Time (min)	Adsorption Heat (J/g)
Mnt	0.6%	112	−11.957
	1.0%	145	−14.029
Kln	0.6%	73	−15.333
	1.0%	90	−24.985

## Data Availability

Not applicable.

## Supplementary Materials

The following are available online at https://www.mdpi.com/article/10.3390/molecules27227950/s1, Figure S1: Schematic of the experimental setup for measuring the interfacial modulus; Figure S2: The adsorption isotherms of (a) Mnt and (b) Kln at 293, 303, and 313 K; Figure S3: SEM images of (a) Mnt and (b) Kln; Figure S4: Sedimentation tests of suspensions with different concentration ratios of clays; Table S1: Structural parameters of different systems at 298 K; Table S2: Force values of the two clays interacting with oil droplets at 298 K.

## Sample Availability

Not available.
