# Supplementary material for: Interfacial Interaction of Clay and Saturates in Petroleum-Contaminated Soil: Effect of Clay Surface Heterogeneity
Source: Molecules. 2022 Nov 17;27(22):7950. doi: 10.3390/molecules27227950 (PMC9692532; doi:10.3390/molecules27227950)
Supplement: Supplementary file 1 [file molecules-27-07950-s001.zip › molecules-2005212-supplementary.pdf]

Article

Interfacial Interaction of Clay and Saturates in Petroleum-Contaminated Soil: Effect of Clay Surface Heterogeneity

Yang Yang [1,2], Xing Liang [1,2] and Xiaobing Li [1,*]

[1] National Center for Coal Preparation and Purification Engineering Research, China University of Mining and Technology, Xuzhou 221116, China
[2] School of Chemical Engineering and Technology, China University of Mining and Technology, Xuzhou 221116, China
* Correspondence: xiaobig.li@cumt.edu.cn

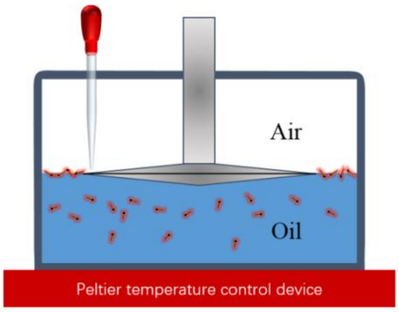

Figure S1. Schematic of the experimental setup for measuring the interfacial modulus.

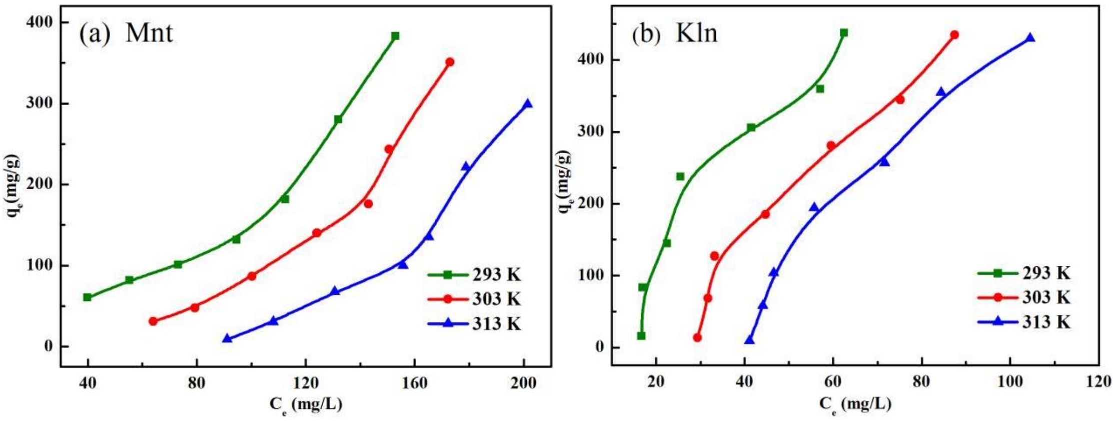

Figure S2. The adsorption isotherms of (a) Mnt and (b) Kln at 293, 303 and 313 K.

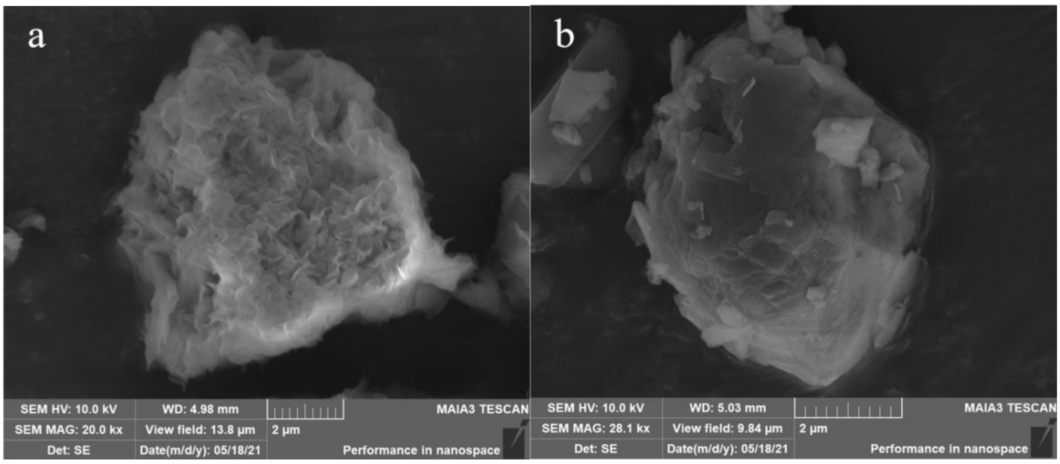

Figure S3. SEM images of (a) Mnt and (b) Kln.

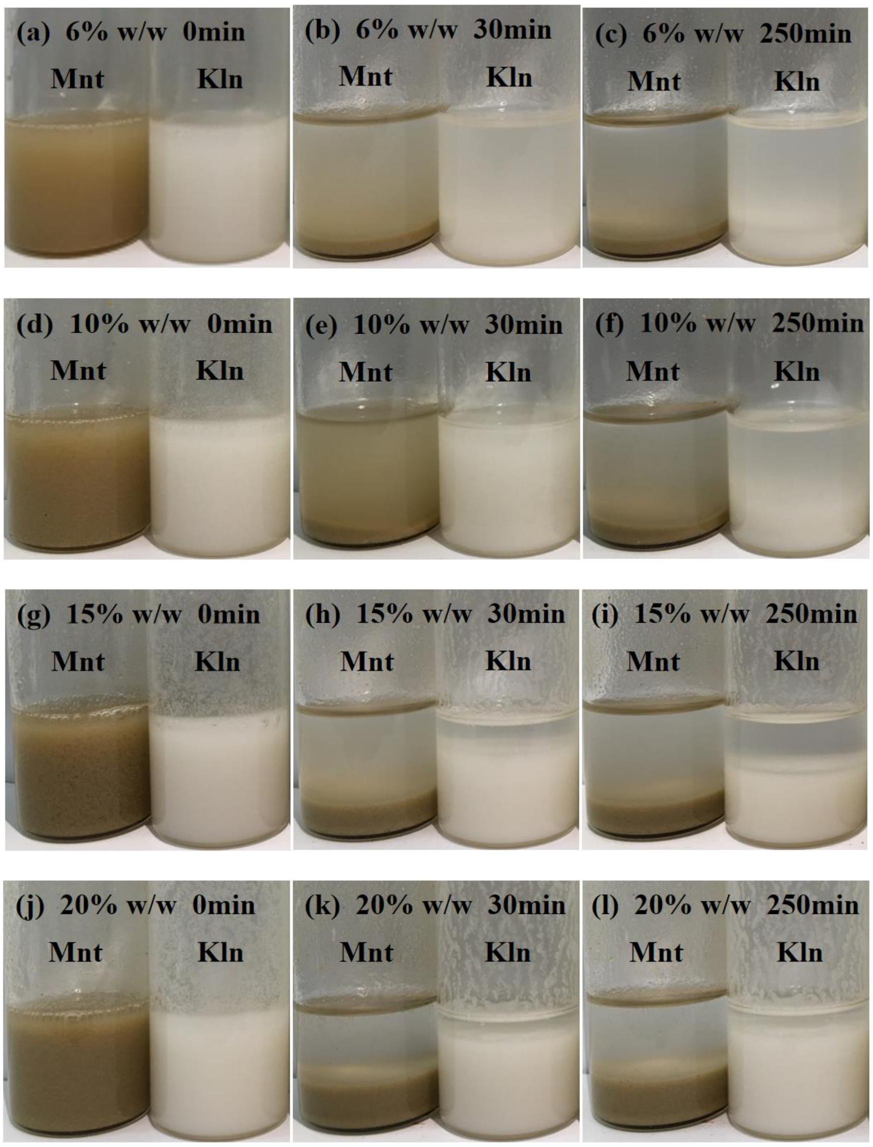

Figure S4. Sedimentation tests of suspensions with different concentration ratios of clays.

Table S1 Structural parameters of different systems in 298 K.

	Initial configuration (a×b×c), Å	Equilibrium configuration (a×b×c), Å	Interfacial angles (α×β×γ), °
Mnt 001 surface	61.20×51.51×131.17	61.20×51.51×131.17	90.00×90.00×75.01
Mnt 010 edge	60.10×51.50×115.71	60.10×51.51×115.71	90.00×90.00×90.00
Kln 001 surface	60.98×51.29×117.86	60.98×51.29×117.86	90.00×90.00×75.02
Kln 010 edge	44.49×51.29×113.65	44.49×51.29×113.65	90.00×90.00×93.37

Table S2. Force values of the two clay interacting with oil droplets at 298 K.

Force, μN	Clay	
	Mnt	Kln
F_{in}	80.87±0.03	96.69±0.02
F_{max}	111.18±0.01	122.65±0.03
F_{off}	22.72±0.05	32.68±0.06
